# Modification of genetic influences on adiposity between 36 and 63 years of age by physical activity and smoking in the 1946 British Birth Cohort Study

**DOI:** 10.1038/nutd.2014.33

**Published:** 2014-09-08

**Authors:** W Johnson, K K Ong, C E Elks, N J Wareham, A Wong, G Muniz-Terrera, R Hardy, on behalf of the MRC NSHD scientific and data collection teams

**Affiliations:** 1 MRC Unit for Lifelong Health and Ageing at UCL, London, UK; 2 MRC Epidemiology Unit, Institute of Metabolic Science, Addenbrooke's Hospital, Cambridge, UK

## Abstract

**Background:**

Previous studies reporting on the interaction between physical activity and genetic susceptibility on obesity have been cross-sectional and have not considered the potential influences of other lifestyle behaviours. The aim of this study was to examine modification of genetic influences on changes across age in adiposity during mid-adulthood by physical activity and smoking.

**Methods:**

The sample comprised 2444 participants who were genotyped for 11 obesity variants and had body mass index (BMI), waist circumference-to-height ratio (WHtR), physical activity and smoking measures at 36, 43, 53 and 60–64 years of age. A genetic risk score (GRS) comprising the sum of risk alleles was computed. Structural equation models investigated modification of the longitudinal GRS associations by physical activity (active versus inactive) and smoking (non-smoker versus smoker), using a latent linear spline to summarise BMI or WHtR (multiplied by 100) at the age of 36 years and their subsequent rates of change over age.

**Results:**

Physical activity at the age of 36 years attenuated the GRS associations with BMI and WHtR at the same age (*P*-interaction 0.009 and 0.004, respectively). Further, physical activity at the age of 53 years attenuated the GRS association with rate of change in BMI between 53 and 63 years of age (by 0.012 kg m^−2^ per year (95% confidence interval (CI): 0.001, 0.024), *P*-interaction 0.004). Conversely, smoking at the age of 43 years showed a trend towards augmenting the GRS association with rate of change in WHtR between 43 and 63 years of age (by 0.012 (95% CI: 0.001, 0.026), *P*-interaction 0.07). Estimated GRS effect sizes were lowest at all ages in the healthiest group (e.g., active non-smokers).

**Conclusions:**

Healthy lifestyle behaviours appeared to attenuate the genetic influence on changes across age in BMI and central adiposity during mid-adulthood. An active lifestyle and not smoking may have additive effects on reducing the genetic susceptibility to obesity in adults.

## Introduction

Genome-wide association studies have identified common genetic variants robustly associated with anthropometric indicators of adiposity.^1, 2, 3, 4, 5, 6, 7^ To date, there are 32 well-established genetic loci for body mass index (BMI) in middle-aged adults of European descent,^1,2,4, 5, 6^ and the strongest loci for BMI are the same as those for indictors of central adiposity such as waist circumference.^3^ Ravussin and Bouchard^8^ have suggested that strong genetic susceptibility to obesity, however, is not ‘unmasked' unless individuals are exposed to obesogenic environments. The pertinent public health question is whether or not an individual can attenuate the influence of their genetic risk for adiposity by healthy lifestyle behaviours?

Physical activity is one of the most promising behavioural candidates for obesity prevention and intervention programmes,^9, 10, 11^ and smoking is a key risk factor for obesity-related chronic disease;^12^ thus, these lifestyle behaviours have been logical targets for gene–environment interaction studies. Most of the publications have investigated how single-nucleotide polymorphisms (SNPs) in or near *FTO*, the strongest genetic susceptibility locus, interact with physical activity to influence BMI and obesity in cross-sectional analyses.^13, 14, 15, 16, 17, 18, 19, 20, 21^ A recent meta-analysis reported that the odds of obesity associated with the *FTO* risk allele was attenuated in active adults compared with inactive adults by 27%, and that there was no such attenuation in children.^17^ The only gene-by-smoking publication in our literature review investigated modification of individual SNP influences on BMI.^22^ Only weak evidence was observed for two SNPs: the influence of rs9939609 (*FTO*) on BMI was attenuated in never/former smokers, but the influence of rs6548238 (*TMEM18*) was accentuated. As smokers tend to have greater central adiposity and actually lower BMI than non-smokers,^23, 24, 25, 26, 27^ a clearer pattern of modification might be expected when using an indicator of central adiposity. Other gaps in the literature comprise (1) lack of knowledge about the combined influences of physical activity and smoking on modifying genetic susceptibility for obesity and (2) a longitudinal perspective to understand whether or not improving physical activity levels or reducing smoking rates will impact on how much adiposity people subsequently accumulate over a substantial period of the life course due to genetic risk.

Using data covering 36 to 60–64 years of age, this is the first paper to investigate modification of a genetic risk score (GRS) that indicates total genetic susceptibility on total body adiposity (indicated by BMI) and central adiposity (indicated by waist circumference-to-height ratio (WHtR)) across adulthood by physical activity and smoking across adulthood. Our aims were (1) to examine how being active versus inactive modifies GRS influences on BMI/WHtR at baseline and subsequent rates of change across age in BMI/WHtR during different periods of adulthood, (2) to examine how being a non-smoker versus smoker modifies GRS influences on the same traits and (3) to investigate the extent to which any modification by physical activity varies according to smoking (and *vice versa*).

## Subjects and methods

### Sample

The Medical Research Council (MRC) National Survey of Health and Development (NSHD) is based on a representative sample of 5362 singletons born to married women in 1 week in March 1946 in England, Scotland and Wales.^28,29^ Data collections have taken place at the age of 36 years in 1982 (*N*=3322), 43 years in 1989 (*N*=3262), 53 years in 1999 (*N*=3035) and 60–64 years in 2006–2010 (*N*=2229). The participants remain broadly representative of native-born British men and women of the same age.^30,31^ At 53 years of age, the majority (*N*=2989) of participants were interviewed and examined in their homes by research nurses, with others completing a postal questionnaire (*N*=46). Contact was not attempted for individuals who refused to take part (*N*=950), were living abroad (*N*=585), were untraced (*N*=316) or had died (*N*=476). Blood samples for DNA extraction were collected from 2756 of the participants who had a home visit. After deducting participants who were not successfully genotyped (*N*=304) and then participants without a measure of BMI or WHtR (*N*=8), the sample of the present study comprised 2444 participants (1220 males and 1224 females).

### Ethics

The study received Multi-Centre Research Ethics Committee approval (07/H1008/168) and informed consent was given by participants.

### Adiposity indicators

Weight, height and waist circumference were measured using standard protocols at 36, 43, 53 and 60–64 years of age; BMI (weight (kg)/height (m)^2^) and WHtR (waist circumference (m)/height (m)) were computed. The WHtR is used because it is a better screening tool than waist circumference or waist circumference-to-hip ratio for cardiometabolic risk factors and because it places both sexes and all ethnic groups on the same scale.^32,33^ Internal *Z*-scores for BMI and WHtR were calculated using the Lambda–Mu–Sigma method.^34^ Overweight was defined as a BMI ⩾25 kg m^−2^ but <30 kg m^−2^, obesity was defined as a BMI ⩾30 kg m^−2^ and central obesity was defined as a WHtR ⩾0.5.

### Genetic risk score

The GRS is the same as that used in a previous MRC NSHD publication in 2012, thereby allowing comparison between publications, and is based on 11 SNPs that had been genotyped at that time.^35^ In Speliotes *et al.*'s paper,^1^ these 11 SNPs accounted for approximately two-thirds of the 1.45% variance in BMI that was explained by all 32 SNPs. Further, the GRS includes the strongest loci for indicators of both total body and central adiposity (i.e., in or near *FTO* and *MC4R*) and as such is suitable for use in the investigation of gene–environment interactions on BMI and WHtR in the present paper.

DNA was extracted and purified from whole blood using the Puregene DNA Isolation Kit (Flowgen, Hessle, UK) according to the manufacturer's protocol.^36^ Rs1421085 (*FTO*) and rs17782313 (*MC4R*) were genotyped by Source Bioscience PLC with the use of Applied Biosystems SNPlex Technology (Applied Biosystems, Foster City, CA, USA), which is based on an Oligonucleotide Ligation Assay combined with multiplex PCR amplification and capillary electrophoresis. Rs6548238 (*TMEM18*), rs8055138 (*SH2B1*), rs11084753 (*KCTD15*), rs10838738 (*MTCH2*), rs2815752 (*NEGR1*), rs925946 (*BDNF*) and rs10913469 (*SEC16B*) were genotyped using the Sequenom iPLEX platform (Sequenom, San Diego, CA, USA), and rs10938397 (*GNPDA2*) and rs7647305 (*ETV5*) were genotyped using custom TaqMan SNP genotyping assays according to the manufacturer's protocol (Applied Biosystems). Call rates for all SNPs were >0.95%, and allelic distributions were in Hardy–Weinberg equilibrium (*P*-values >0.05).

For each of the 11 SNPs, the allele known to be associated with obesity was considered the risk allele and then the number of risk alleles for each SNP (0, 1 or 2) was counted. The GRS was computed for each participant as the summation of risk alleles across the 11 SNPs, so that a one-unit increase corresponded to an increase of one risk allele. The GRS was normally distributed and centred about the mean of 12 risk alleles. A separate weighted GRS was similarly computed by first multiplying the number of risk alleles for each SNP by the appropriate estimated risk allele effect size on BMI from Table 1 in Speliotes *et al.*'s^1^ paper before summation. The weighted GRS was tested in sensitivity analyses for BMI, but these sensitivity analyses for WHtR were not conducted owing to a lack of established SNP effects sizes on WHtR.

### Lifestyle behaviours

Participation in leisure time physical activity was ascertained at 36, 43, 53 and 60–64 years of age during interviews with research nurses. At age 36 of years, this was based on the Minnesota leisure time physical activity questionnaire on the frequency and duration of participation in 27 different activities in the preceding month.^37,38^ At 43, 53 and 60–64 years of age, this was based on more basic questions about participation in any sports and vigorous leisure time activities or exercises.^39^ At 43 years of age, participants reported how many months in the preceding year and how often in these months each activity was performed. At 53 and 60–64 years of age, participants reported the number of occasions on which activities were undertaken in the preceding 4 weeks. For the purpose of this study, physical activity at each age was categorised as ‘inactive' if the participant reported no participation or ‘active' if they reported participating at least once in the previous month (aged 36 years), at least once per month during the 1-year recall period (aged 43 years), or at least once in the previous 4 weeks (aged 53 and 60–64 years). Smoking status was also reported at each age and participants were categorised as being a ‘smoker' if they currently smoked or a ‘non-smoker' if they had never smoked or were an ex-smoker. Further, a composite variable at each age with the categories ‘inactive/smoker', ‘active/smoker', ‘inactive/non-smoker' and ‘active/non-smoker' was computed.

### Statistical analysis

BMI and WHtR *Z*-scores at each age were regressed on GRS to confirm that it was measuring genetic risk for adiposity in our sample. In addition, BMI and WHtR *Z*-scores were regressed on concurrent physical activity and separately on concurrent smoking to confirm that the associations were in the expected directions.

Two structural equation models (SEMs), one for BMI and one for WHtR, were built to address each study aim. Each SEM summarised the longitudinal BMI or WHtR (multiplied by 100 for presentation purposes) data as a latent linear spline with one knot. The knot was positioned at the age of 53 years for BMI, thus the latent spline parameters were (1) an intercept representing size at the age of 36 years, (2) a first slope representing rate of change from 36 to 53 years of age and (3) a second slope representing rate of change from 53 to 63 years of age. The knot was positioned at the age of 43 years for WHtR, so the latent spline parameters were (1) an intercept representing size at the age of 36 years, (2) a first slope representing rate of change from 36 to 43 years of age and (3) a second slope representing rate of change from 43 to 63 years of age.

**Figure 1:**
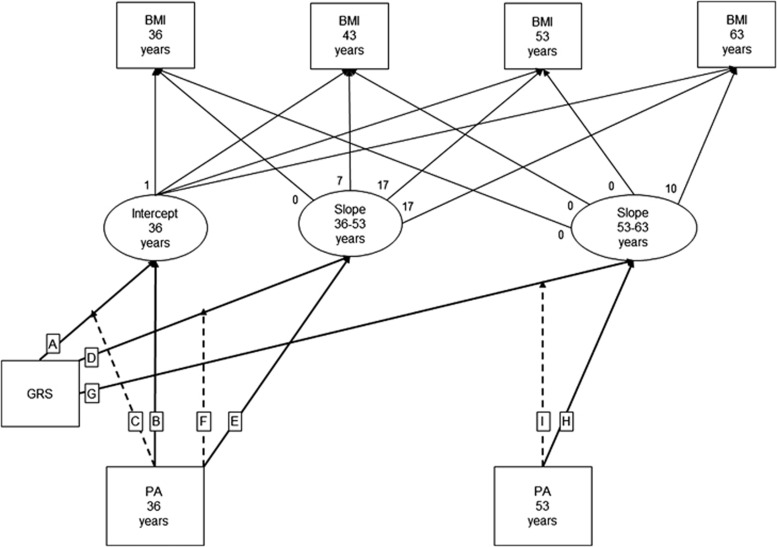
A latent linear spline SEM to test modification of GRS associations with BMI trajectories by physical activity (PA). GRS was computed for each individual as the summation of risk alleles across 11 obesity variants and PA was ascertained at 36 and 53 years of age during interviews with a research nurse. Leisure time PA assessment was based on the Minnesota leisure time PA questionnaire at the age of 36 years and on more basic questions at the age of 53 years. At each age, participants who reported no leisure time PA were classified as ‘inactive' and those who reported any relevant activity (in the previous month at the age of 36 years and in the previous 4 weeks at the age of 53 years) were classified as ‘active'. Thin arrows are used for the latent linear spline that summarises the serial BMI data for each individual as an intercept (i.e., kg m^−2^ at the age of 36 years) and two slope terms (i.e., kg m^−2^ per year between 36 and 53 and between 53 and 63 years of age). Solid thick arrows are used for main associations of GRS and PA with the intercept and slope terms. Dashed thick arrows are used for GRS-by-PA interaction associations with the intercept and slope terms. Each thick arrow is labelled with a letter that corresponds to an estimated parameter in Table 2.

In each SEM, (1) the latent intercept is regressed on sex and GRS-by-the lifestyle behaviour variable (and the components of this interaction) at the age of 36 years to test for modification of GRS influences on size at baseline by the lifestyle behaviour variable at baseline, conditional on sex; (2) the first latent slope is regressed on sex and GRS-by-the lifestyle behaviour variable (and the components of this interaction) at the age of 36 years to test for modification of GRS influences on rate of change before the knot by the lifestyle behaviour variable at baseline (conditional on sex); and (3) the second latent slope is regressed on sex and GRS-by-the lifestyle behaviour variable (and the components of this interaction) at the knot to test for modification of GRS influences on rate of change after the knot by the lifestyle behaviour variable at the knot (conditional on sex). Figure 1 provides a visual representation of the SEM for BMI and physical activity. The SEMs were estimated in Mplus^40^ and were used to estimate GRS effect sizes at each age within each physical activity or/and smoking group and these were plotted.

In secondary analyses, SEMs including a GRS-by-physical activity-by-smoking interaction term were used to formally test whether or not any modification by physical activity varied according to smoking (or *vice versa*). Sensitivity analyses included SEMs stratified by sex, SEMs stratified by weight status at the age of 36 years (i.e., normal weight or overweight or obese) or central obesity status at the age of 36 years (i.e., no central obesity or central obesity), and for BMI an SEM that included physical activity or/and smoking at 43 years of age instead of 53 years of age. Further, the SEMs for BMI were re-run using the weighted GRS to confirm that constraining each SNP to have an equal contribution to genetic risk in the main analyses was not inappropriate.

## Results

Both BMI and WHtR increased across adulthood so that by 60–64 years of age 72% of the sample was overweight or obese and 86% had central obesity (Table 1). The percentage of participants categorised as active decreased from 64% at the age of 36 years to 35% at the age of 60–64 years, whereas the percentage of smokers decreased from 32% at the age of 36 years to 12% at the age of 60–64 years.

The GRS was positively associated with BMI and WHtR *Z*-scores at all ages (data not shown). Further, active participants tended to have lower BMI and WHtR *Z*-scores than inactive participants, and non-smokers tended to have higher BMI but lower WHtR *Z*-scores than smokers (data not shown).

Using the SEM for BMI and physical activity as an example, Figure 1 is a visual representation of the model and Table 2 gives the corresponding parameter estimates (labelled A-I). At 36 years of age, the GRS and BMI were positively associated (A) and the active group was estimated to have a lower mean BMI than the inactive group (B). Further, the estimated GRS effect size on BMI was 0.189 kg m^−2^ (95% confidence interval (CI): 0.047, 0.331) lower in the active group compared with the inactive group (C). There was no such effect modification on rate of change in BMI between 36 and 53 years of age (F), but the GRS association with rate of change in BMI between 53 and 63 years of age was 0.012 kg m^−2^ per year (95% CI: 0.001, 0.024) lower in participants who were active at age 53 years of age compared with those who were inactive (I).

**Figure 2:**
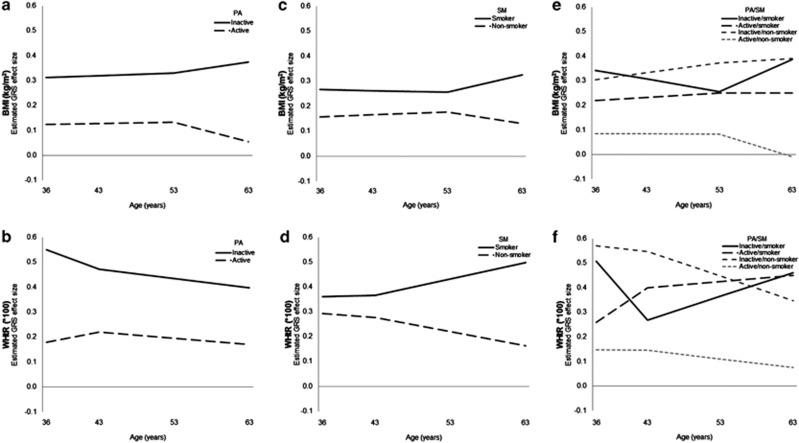
Estimated GRS effect sizes within each physical activity (PA) or/and smoking (SM) group. GRS was computed for each individual as the summation of risk alleles across 11 obesity variants, PA was ascertained at the age of 36 years and either at the age of 53 years (for BMI) or 43 years (for WHtR) during interviews with a research nurse, SM was ascertained at the age of 36 years and either at the age of 53 years (for BMI) or 43 years (for WHtR) during interviews with a research nurse. Participants were categorised as being a ‘smoker' if they currently smoked or a ‘non-smoker' if they had never smoked or were an ex-smoker. The GRS effect sizes in panels **a** and **b** are estimated from the models shown in Supplementary Table 1, the GRS effect sizes in panels **c** and **d** are estimated from the models shown in Supplementary Table 2 and the GRS effect sizes in panels **e** and **f** are estimated from the models shown in Supplementary Table 3. Results for participants who changed group (e.g., active at the age of 36 years but inactive at the age of 53 years) are not shown because this was not explicitly modelled (i.e., there was no parameter for participants who changed groups). However, it is implicit that the trajectories for individuals who changed group would diverge at the age of 53 years (for BMI) or 43 years (for WHtR) from the trajectory of the old group (e.g., active) to follow the gradient of the trajectory for the new group (e.g., inactive).

The parameter estimates in Table 2 were used to estimate GRS effect sizes at each age (i.e., 36, 43, 53 and 63 years) within each physical activity group, and these results are shown in Figure 2a. The estimated GRS effect sizes on BMI were consistently lower in active compared with inactive participants. At 36 years of age, the difference between the two groups of 0.189 kg m^−2^ corresponds to the estimate of the GRS-by-physical activity interaction on the intercept in Table 2. Similarly, the widening of differences in estimated GRS effect sizes between the two groups after 53 years of age occurs at 0.012 kgm^−2^ per year, corresponding to the estimate of the GRS-by-physical activity interaction on the slope between 53 and 63 years of age in Table 2.

Figure 2 also shows the pertinent results for the other SEMs; the parameter estimates and model fit statistics for all SEMs can be found in Supplementary Tables 1. Overall model fits showed that each hypothesised SEM was different to a fully saturated model (*P*-values <0.001). With a sample size of over 2000, this was to be expected because of the large power to detect even minor discrepancies. The other fit statistics indicated good to excellent model fit because all values were >0.95.^41^

The estimated GRS effect sizes on WHtR (multiplied by 100) were consistently lower in active compared with inactive participants (Figure 2b). The magnitude of this difference, estimated to be 0.370 (95% CI: 0.116, 0.625) at 36 years of age, remained relatively constant across the age range studied.

For smoking (Figures 2c and d), the estimated GRS effect sizes were lower in non-smokers compared with smokers, but the differences were not nominally significant at *P*-value <0.05 at 36 years of age. Over age, the estimated GRS effect sizes continued to widen between non-smokers and smokers on BMI (between 53 and 63 years of age; interaction *P*-value 0.1) and WHtR (between 43 and 63 years of age; interaction *P*-value 0.07).

Considering the combination of physical activity and smoking (Figure 2e and f), the estimated GRS effect sizes were lowest in the healthiest lifestyle group (e.g., active/non-smokers). For BMI, the estimated GRS effect size at 36 years of age was 0.258 kg m^−2^ (95% CI: 0.037, 0.480) lower in active/non-smokers compared with the least healthy lifestyle group (e.g., inactive/smokers), and this difference widened between 53 and 63 years of age by 0.022 kg m^−2^ per year (95% CI: 0.003, 0.042). However, GRS-by-physical activity-by-smoking interaction terms in secondary SEMs were not nominally significant (all *P*-values >0.2) (data not shown), thereby indicating that the combined modifying effects of physical activity and smoking on GRS effect sizes were not more than additive.

Sensitivity analyses did not produce any estimates that were fundamentally different to those reported here from the main analyses (data not shown).

## Discussion

This study provides novel longitudinal evidence in adulthood that an active lifestyle may attenuate total body adiposity (as indicated by BMI) accumulation due to genetic risk; tentative novel evidence was also found to suggest that another healthy lifestyle factor, not smoking, may attenuate central adiposity (as indicated by WHtR) accumulation due to genetic risk.

Other studies of gene–environment interaction on adiposity traits were largely cross-sectional and typically considered only one lifestyle behaviour.^14, 15, 16, 17, 18, 19, 20, 21, 22,42, 43, 44, 45, 46^ The present study is the first to test whether the level of attenuation achieved by one lifestyle behaviour varies across different groups of a second. Three-way GRS-by-physical activity-by-smoking interaction terms were not nominally significant, thereby indicating no evidence for departure from additive effects in the level of attenuation achieved by physical activity and not smoking.

The meta-analysis of Kilpelainen *et al.*^17^ reported that the effect size on BMI related to each risk allele in rs9939609 (*FTO*) was 0.14 kg m^−2^ lower in physically active adults compared with physically inactive adults. Similarly, we found that the effect size on BMI related to each risk allele increase in GRS corresponded to a BMI increase of 0.31 kg m^−2^ at 36 years of age, but that being active attenuated this association by 0.19 kg m^−2^. This crudely equates to a remarkable 60% reduction in genetic influences on BMI.

Few studies have investigated gene–environment interactions on indicators of adiposity other than BMI.^8,16,34^ Our WHtR results strengthen the limited evidence that physical activity modifies genetic influences on central adiposity as well as total body adiposity.^17^ They also provide the first tentative evidence that not smoking attenuates genetic influences on central adiposity accumulation. The paradox of lower BMI but higher waist circumference and waist-to-hip ratio in smokers compared with non-smokers has been well documented.^23, 24, 25, 26, 27,47^ Various mechanisms have been proposed to explain the greater central obesity of smokers compared with non-smokers, including higher fasting plasma cortisol concentrations in response to stimulation of sympathetic nervous system activity.^48,49^ Our results suggest that augmented genetic effects may also have a role.

The mechanisms by which lifestyle behaviours modify the influences of obesity genetic variants are not fully understood. Epigenetic regulation of gene expression is a likely pathway because epigenetic markers, such as DNA methylation, can be modified by environmental factors^50, 51, 52^ and are associated with at least the strongest genetic susceptibility locus for obesity.^53, 54, 55^ The advent of high-throughput DNA methylation bead chip technology will allow large cohort studies to better quantify complex interactions between genotype, environment and DNA methylation variation in the near future.^56,57^

A particular strength of the present study is the robust analysis of longitudinal data covering 36 to 60–64 years of age. Our results build on cross-sectional gene–environment interaction on obesity studies and one published longitudinal study that found annual change in BMI, over an average follow-up of 3.6 years, to be attenuated by approximately 0.006 kg m^−2^ per year per risk allele (in a 12 SNP GRS) in a group who were active at baseline compared with a group who were inactive at baseline (aged 39–79 years).^45^ What we add to this literature is the knowledge that healthy lifestyle behaviours may be important in the determination of how much adiposity is gained, because of genetic risk, over a substantial period of adulthood.

In terms of limitations, physical activity and smoking status were self-reported, which might have biased estimates if there was systematic under- or over-reporting, the questions used to assess physical activity changed over age and the GRS explained only a small fraction of the variance in BMI/WHtR. The SEMs efficiently handled missing BMI and WHtR data, but the missing physical activity and smoking data did have the potential to introduce some bias. The estimated relationships of physical activity/smoking with BMI/WHtR might be confounded by other lifestyle behaviours and failure to adjust for these might have biased the GRS-by-physical activity/smoking estimates. Differences between physical activity groups in the influence of genetic susceptibility to adiposity were already present at 36 years of age, but lifestyle behaviour data before 36 years of age are limited in the MRC NSHD, making it impossible to determine the age when physical activity started to modify the influence of genetic susceptibility to adiposity. Finally, the sample comprised individuals who were all born in 1946, which makes it impossible to distinguish between age effects and period effects, and may limit generalisability of results to the modern-day British population of adults who were born more recently than in 1946. Other studies have shown that the positive effect of obesity genetic variants on adulthood adiposity has strengthened over successive cohorts,^56^ which suggests that the scope for attenuation of genetic influences on adiposity by lifestyle behaviours may be greater in more recently born cohorts.

In conclusion, we provide novel evidence that being active in mid-adulthood attenuates genetic influences on subsequent adiposity accumulation, and that being a non-smoker in mid-adulthood may attenuate genetic influences on subsequent central adiposity accumulation. The public health message is clear; some individuals may be more susceptible to obesity than others, but this can at least be partly offset by remaining active across the life course and perhaps not smoking. Research in larger samples is needed to confirm whether or not the combined modifying effect of lifestyle behaviours is more than additive.

## Figures and Tables

**Table 1:** Description of study sample of 2444 adults

	*36 Years*	*43 Years*	*53 Years*	*60–64 Years*
Exact age (years), median (range)	36.3 (36.0, 37.2)	43.5 (42.8, 44.3)	53.5 (53.0, 54.2)	63.3 (59.8, 64.9)
				
*BMI (kg m*^−*2*^*), mean (s.d.)*	24.1 (3.5)	25.4 (4.0)	27.4 (4.6)	28.0 (4.9)
Normal weight^a^, *N* (%) (valid %)^b^	1457 (59.6) (66.0)	1203 (49.2) (52.5)	793 (32.5) (32.7)	501 (20.5) (28.3)
Overweight^a^, *N* (%) (valid %)^b^	629 (25.7) (28.5)	825 (33.8) (36.0)	1071 (43.8) (44.1)	756 (30.9) (42.7)
Obese^a^, *N* (%) (valid %)^b^	123 (5.0) (5.6)	264 (10.8) (11.5)	564 (23.1) (23.2)	514 (21.0) (29.0)
Missing, *N* (%)	235 (9.6)	152 (6.2)	16 (0.7)	673 (27.5)
				
				
*WHtR, mean (s.d.)*	0.49 (0.06)	0.50 (0.07)	0.54 (0.07)	0.58 (0.07)
No central obesity^c^, *N* (%) (valid %)^b^	1295 (53.0) (58.5)	1207 (49.4) (52.8)	660 (27.0) (27.1)	249 (10.2) (14.1)
Central obesity^c^, *N* (%) (valid %)^b^	917 (37.5) (41.5)	1079 (44.1) (47.2)	1772 (72.5) (72.9)	1517 (62.1) (85.9)
Missing, *N* (%)	232 (9.5)	158 (6.5)	12 (0.5)	678 (27.7)
				
*PA*^d^				
Inactive, *N* (%) (valid %)^b^	804 (32.9) (36.2)	1191 (48.7) (51.5)	1189 (48.6) (48.7)	1138 (46.6) (65.3)
Active, *N* (%) (valid %)^b^	1414 (57.9) (63.8)	1120 (45.8) (48.5)	1253 (51.3) (51.3)	606 (24.8) (34.7)
Missing, *N* (%)	226 (9.2)	133 (5.4)	2 (0.1)	700 (28.6)
				
*SM*^d^				
Smoker, *N* (%) (valid %)^b^	719 (29.4) (32.3)	665 (27.2) (28.9)	595 (24.3) (24.3)	237 (9.7) (12.7)
Non-smoker, *N* (%) (valid %)^b^	1505 (61.6) (67.7)	1640 (67.1) (71.1)	1848 (75.6) (75.6)	1636 (66.9) (87.3)
Missing, *N* (%)	220 (9.0)	139 (5.7)	1 (0.0)	571 (23.4)
				
*PA/SM*^d^				
Inactive/smoker, *N* (%) (valid %)^b^	301 (12.3) (13.6)	425 (17.4) (18.4)	386 (15.8) (15.8)	157 (6.4) (9.7)
Active/smoker, *N* (%) (valid %)^b^	415 (17.0) (18.7)	240 (9.8) (10.4)	209 (8.6) (8.6)	35 (1.4) (2.2)
Inactive/non-smoker, *N* (%) (valid %)^b^	503 (20.6) (22.7)	762 (31.2) (33.1)	803 (32.9) (32.9)	889 (36.6) (55.0)
Active/non-smoker, *N* (%) (valid %)^b^	998 (40.8) (45.0)	878 (35.9) (38.1)	1044 (42.7) (42.8)	534 (21.8) (33.1)
Missing, *N* (%)	227 (9.3)	139 (5.7)	2 (0.1)	829 (33.9)

Abbreviations: BMI, body mass index; PA, physical activity; SM, smoking; WHtR, waist circumference-to-height ratio.

a Normal weight: BMI <25 kg m^−2^; overweight: BMI ⩾25 kg m^−2^, but <30 kg m^−2^; obese: BMI ⩾30 kg m^−2^.

b Valid % excludes missing data and is calculated as the *N* in that cell divided by the *N* who had data for that particular variable at that particular age multiplied by 100.

c No central obesity: WHtR <0.5; central obesity: WHtR ⩾0.5.

d Participation in leisure time PA and smoking status were ascertained at each age during an interview with a research nurse. Participants were categorised as being a ‘smoker' if they currently smoked or a ‘non-smoker' if they had never smoked or were an ex-smoker. Leisure time PA assessment was based on the Minnesota leisure time PA questionnaire at 36 years of age and on more basic questions at 43, 53 and 60–64 years of age. At each age, participants who reported no leisure time physical activity were classified as ‘inactive' and those who reported any relevant activity (in the previous month at 36 years of age, in the previous year at 43 years of age and in the previous 4 weeks at 53 and 60–64 years of age) were classified as ‘active'.

**Table 2:** Latent linear spline SEM to test modification of GRS associations with BMI trajectories by physical activity

		*BMI (kg* *m*^−*2*^), N*=2216*
	*Parameter*^a^	B *(95% CI),* P*-value*
*Intercept*		*Size 36 years*
GRS (per risk allele)^b^	A	0.312 (0.196, 0.428), <0.001
PA at age of intercept^c^		
Inactive (referent)		−−
Active	B	−0.813 (−1.113, −0.512), <0.001
GRS-by-PA at age of intercept		
Inactive (referent)		−−
Active	C	−0.189 (−0.331, −0.047), 0.009
		
*Slope 1*		*Change per year 36–53 years*
GRS (per risk allele)^b^	D	0.001 (−0.005, 0.007), 0.7
PA at age of intercept^c^		
Inactive (referent)		−−
Active	E	0.004 (−0.011, 0.019), 0.6
GRS-by-PA at age of intercept		
Inactive (referent)		−−
Active	F	0.000 (−0.008, 0.007), 0.9
		
*Slope 2*		*Change per year 53–63 years*
GRS (per risk allele)^b^	G	0.005 (−0.004, 0.014), 0.3
PA at age of knot^c^		
Inactive (referent)		−−
Active	H	−0.018 (−0.043, 0.008), 0.2
GRS-by-PA at age of knot		
Inactive (referent)		−−
Active	I	−0.012 (−0.024, −0.001), 0.04

Abbreviations: BMI, body mass index; GRS, genetic risk score; PA, physical activity; SEM, structural equation model.

a Each parameter is labelled with a letter that corresponds to a thick arrow in the visual representation of this SEM in Figure 1.

b Computed for each individual as the summation of risk alleles across 11 obesity variants.

c Participation in leisure time PA was ascertained at each age during an interview with a research nurse. Leisure time PA assessment was based on the Minnesota leisure-time physical activity questionnaire at 36 years of age and on more basic questions at 53 years of age. At each age, participants who reported no leisure time PA were classified as ‘inactive' and those who reported any relevant activity (in the previous month at 36 years of age and in the previous 4 weeks at 53 years of age) were classified as ‘active'.
