# Supplementary material for: Modification of genetic influences on adiposity between 36 and 63 years of age by physical activity and smoking in the 1946 British Birth Cohort Study
Source: Nutr Diabetes. 2014 Sep 8;4(9):e136–. doi: 10.1038/nutd.2014.33 (PMC4183974; doi:10.1038/nutd.2014.33)
Supplement: Supplementary Tables [file nutd201433x1.doc]

**Supplementary Table 1. Latent linear spline SEMs testing modification of GRS associations with adiposity trajectories by physical activity**

|  | **BMI (kg/m^2^)**  **N = 2216** | **WHtR (*100)**  **N = 2142** |
| --- | --- | --- |
|  | **B (95% CI) P-value** | |
| **Intercept** | **Size**  **36 years** | **Size**  **36 years** |
| GRS (per risk allele)^1^ | 0.312 (0.196, 0.428) <0.001 | 0.550 (0.343, 0.758) <0.001 |
| PA at age of intercept^2^ |  |  |
| Inactive (referent) | -- | -- |
| Active | -0.813 (-1.113, -0.512) <0.001 | -1.930 (-2.470, -1.390) <0.001 |
| GRS-by-PA at age of intercept |  |  |
| Inactive (referent) | -- | -- |
| Active | -0.189 (-0.331, -0.047) 0.009 | -0.370 (-0.625, -0.116) 0.004 |
| **Slope 1** | **Change per year**  **36-53 years** | **Change per year**  **36-43 years** |
| GRS (per risk allele)^1^ | 0.001 (-0.005, 0.007) 0.7 | -0.011 (-0.035, 0.013) 0.4 |
| PA at age of intercept^2^ |  |  |
| Inactive (referent) | -- | -- |
| Active | 0.004 (-0.011, 0.019) 0.6 | 0.047 (-0.014, 0.109) 0.1 |
| GRS-by-PA at age of intercept |  |  |
| Inactive (referent) | -- | -- |
| Active | 0.000 (-0.008, 0.007) 0.9 | 0.017 (-0.012, 0.046) 0.3 |
| **Slope 2** | **Change per year**  **53-63 years** | **Change per year**  **43-63 years** |
| GRS (per risk allele)^1^ | 0.005 (-0.004, 0.014) 0.3 | -0.004 (-0.012, 0.005) 0.4 |
| PA at age of knot^2^ |  |  |
| Inactive (referent) | -- | -- |
| Active | -0.018 (-0.043, 0.008) 0.2 | -0.007 (-0.032, 0.017) 0.6 |
| GRS-by-PA at age of knot |  |  |
| Inactive (referent) | -- | -- |
| Active | -0.012 (-0.024, -0.001) 0.04 | 0.001 (-0.010, 0.013) 0.8 |
| **Model fit** |  |  |
| Chi Square |  |  |
| Value | 49 | 60 |
| Degrees of freedom | 15 | 14 |
| P-value | <0.001 | <0.001 |
| 1-RMSEA | 0.968 | 0.961 |
| CFI | 0.996 | 0.992 |
| TFI | 0.991 | 0.982 |

SEM: structural equation model, GRS: genetic risk score, BMI: body mass index, WHtR: waist circumference to height ratio, PA: physical activity, RMSEA: root mean square error of approximation, CFI: comparative fit index, TFI: Tucker-Lewis index

^1^Computed for each individual as the summation of risk alleles across 11 obesity variants.

^2^Participation in leisure time physical activity was ascertained at each age during an interview with a research nurse. Leisure time physical activity assessment was based on the Minnesota leisure-time physical activity questionnaire at age 36 years and on more basic questions at ages 43 and 53 years. At each age, participants who reported no leisure time physical activity were classified as “inactive” and those who reported any relevant activity (in the previous month at age 36 years, in the previous year at age 43 years, and in the previous four weeks at age 53 years) were classified as “active”.

**Supplementary Table 2. Latent linear spline SEMs testing modification of GRS associations with adiposity trajectories by smoking**

|  | **BMI (kg/m^2^)**  **N = 2223** | **WHtR (*100)**  **N = 2142** |
| --- | --- | --- |
|  | **B (95% CI) P-value** | |
| **Intercept** | **Size**  **36 years** | **Size**  **36 years** |
| GRS (per risk allele)^1^ | 0.267 (0.146, 0.388) <0.001 | 0.360 (0.136, 0.583) 0.002 |
| SM at age of intercept^2^ |  |  |
| Smoker (referent) | -- | -- |
| Non-smoker | 0.129 (-0.179, 0.438) 0.4 | -0.199 (-0.762, 0.364) 0.5 |
| GRS-by-SM at age of intercept |  |  |
| Smoker (referent) | -- | -- |
| Non-smoker | -0.109 (-0.255, 0.037) 0.1 | -0.066 (-0.333, 0.200) 0.6 |
| **Slope 1** | **Change per year**  **36-53 years** | **Change per year**  **36-43 years** |
| GRS (per risk allele)^1^ | -0.001 (-0.007, 0.006) 0.8 | 0.001 (-0.024, 0.026) 0.9 |
| SM at age of intercept^2^ |  |  |
| Smoker (referent) | -- | -- |
| Non-smoker | -0.002 (-0.018, 0.014) 0.8 | -0.101 (-0.165, -0.038) 0.002 |
| GRS-by-SM at age of intercept |  |  |
| Smoker (referent) | -- | -- |
| Non-smoker | 0.002 (-0.006, 0.009) 0.6 | -0.003 (-0.033, 0.027) 0.8 |
| **Slope 2** | **Change per year**  **53-63 years** | **Change per year**  **43-63 years** |
| GRS (per risk allele)^1^ | 0.007 (-0.006, 0.020) 0.3 | 0.007 (-0.005, 0.018) 0.3 |
| SM at age of knot^2^ |  |  |
| Smoker (referent) | -- | -- |
| Non-smoker | -0.053 (-0.085, -0.022) 0.001 | -0.044 (-0.072, -0.015) 0.003 |
| GRS-by-SM at age of knot |  |  |
| Smoker (referent) | -- | -- |
| Non-smoker | -0.012 (-0.026, 0.003) 0.1 | -0.012 (-0.026, 0.001) 0.07 |
| **Model fit** |  |  |
| Chi Square |  |  |
| Value | 103 | 44 |
| Degrees of freedom | 15 | 14 |
| P-value | <0.001 | <0.001 |
| 1-RMSEA | 0.950 | 0.968 |
| CFI | 0.989 | 0.995 |
| TFI | 0.977 | 0.989 |

SEM: structural equation model, GRS: genetic risk score, BMI: body mass index, WHtR: waist circumference to height ratio, SM: smoking, RMSEA: root mean square error of approximation, CFI: comparative fit index, TFI: Tucker-Lewis index

^1^Computed for each individual as the summation of risk alleles across 11 obesity variants.

^2^Smoking status was ascertained at each age during an interview with a research nurse. Participants were categorised as being a “smoker” if they currently smoked or a “non-smoker” if they had never smoked or were an ex-smoker.

**Supplementary Table 3. Latent linear spline SEMs testing modification of GRS associations with adiposity trajectories by combined physical activity and smoking groups**

|  | **BMI (kg/m^2^)**  **N = 2215** | **WHtR (*100)**  **N = 2137** |
| --- | --- | --- |
|  | **B (95% CI) P-value** | |
| **Intercept** | **Size**  **36 years** | **Size**  **36 years** |
| GRS (per risk allele)^1^ | 0.342 (0.144, 0.541) 0.001 | 0.507 (0.140, 0.874) 0.007 |
| PA/SM at age of intercept^2^ |  |  |
| Inactive/smoker (referent) | -- | -- |
| Active/smoker | -0.256 (-0.767, 0.255) 0.3 | -1.252 (-2.188, -0.316) 0.009 |
| Inactive/non-smoker | 0.731 (0.240, 1.221) 0.003 | 0.606 (-0.286, 1.499) 0.2 |
| Active/non-smoker | -0.403 (-0.846, 0.039) 0.07 | -1.677 (-2.487, -0.868) <0.001 |
| GRS-by-PA/SM at age of intercept |  |  |
| Inactive/smoker (referent) | -- | -- |
| Active/smoker | -0.123 (-0.373, 0.127) 0.3 | -0.248 (-0.707, 0.211) 0.3 |
| Inactive/non-smoker | -0.038 (-0.283, 0.206) 0.8 | 0.064 (-0.381, 0.510) 0.8 |
| Active/non-smoker | -0.258 (-0.480, -0.037) 0.02 | -0.359 (-0.765, 0.047) 0.08 |
| **Slope 1** | **Change per year**  **36-53 years** | **Change per year**  **36-43 years** |
| GRS (per risk allele)^1^ | -0.005 (-0.015, 0.005) 0.3 | -0.034 (-0.076, 0.008) 0.1 |
| PA/SM at age of intercept^2^ |  |  |
| Inactive/smoker (referent) | -- | -- |
| Active/smoker | 0.001 (-0.025, 0.027) 0.9 | 0.027 (-0.079, 0.133) 0.6 |
| Inactive/non-smoker | -0.007 (-0.032, 0.018) 0.6 | -0.138 (-0.240, -0.037) 0.008 |
| Active/non-smoker | 0.000 (-0.023, 0.022) >0.9 | -0.065 (-0.157, 0.028) 0.2 |
| GRS-by-PA/SM at age of intercept |  |  |
| Inactive/smoker (referent) | -- | -- |
| Active/smoker | 0.007 (-0.006, 0.020) 0.3 | 0.054 (0.002, 0.106) 0.04 |
| Inactive/non-smoker | 0.009 (-0.003, 0.022) 0.2 | 0.031 (-0.020, 0.081) 0.2 |
| Active/non-smoker | 0.005 (-0.006, 0.016) 0.4 | 0.034 (-0.013, 0.080) 0.2 |
| **Slope 2** | **Change per year**  **53-63 years** | **Change per year**  **43-63 years** |
| GRS (per risk allele)^1^ | 0.013 (-0.005, 0.031) 0.2 | 0.010 (-0.005, 0.024) 0.2 |
| PA/SM at age of knot^2^ |  |  |
| Inactive/smoker (referent) | -- | -- |
| Active/smoker | -0.024 (-0.081, 0.033) 0.4 | 0.047 (-0.003, 0.096) 0.06 |
| Inactive/non-smoker | -0.059 (-0.102, -0.016) 0.007 | -0.018 (-0.055, 0.019) 0.3 |
| Active/non-smoker | -0.067 (-0.109, -0.026) 0.001 | -0.034 (-0.071, 0.002) 0.06 |
| GRS-by-PA/SM at age of knot |  |  |
| Inactive/smoker (referent) | -- | -- |
| Active/smoker | -0.013 (-0.039, 0.013) 0.3 | -0.007 (-0.031, 0.017) 0.6 |
| Inactive/non-smoker | -0.011 (-0.032, 0.009) 0.3 | -0.020 (-0.037, -0.002) 0.03 |
| Active/non-smoker | -0.022 (-0.042, -0.003) 0.03 | -0.013 (-0.030, 0.004) 0.1 |
| **Model fit** |  |  |
| Chi Square |  |  |
| Value | 169 | 92 |
| Degrees of freedom | 35 | 34 |
| P-value | <0.001 | <0.001 |
| 1-RMSEA | 0.958 | 0.972 |
| CFI | 0.983 | 0.990 |
| TFI | 0.969 | 0.981 |

SEM: structural equation model, GRS: genetic risk score, BMI: body mass index, WHtR: waist circumference to height ratio, PA: physical activity, SM: smoking, RMSEA: root mean square error of approximation, CFI: comparative fit index, TFI: Tucker-Lewis index

^1^Computed for each individual as the summation of risk alleles across 11 obesity variants.

^2^Participation in leisure time physical activity and smoking status were ascertained at each age during an interview with a research nurse. Participants were categorised as being a “smoker” if they currently smoked or a “non-smoker” if they had never smoked or were an ex-smoker. Leisure time physical activity assessment was based on the Minnesota leisure-time physical activity questionnaire at age 36 years and on more basic questions at ages 43 and 53 years. At each age, participants who reported no leisure time physical activity were classified as “inactive” and those who reported any relevant activity (in the previous month at age 36 years, in the previous year at age 43 years, and in the previous four weeks at age 53 years) were classified as “active”.
